# Human cDC1s display constitutive activation of the UPR sensor IRE1

**DOI:** 10.1002/eji.202149774

**Published:** 2022-04-22

**Authors:** Paulina García‐González, Dominique Fernández, Diane Gutiérrez, Mauro Parra‐Cordero, Fabiola Osorio

**Affiliations:** ^1^ Laboratory of Immunology and Cellular Stress Immunology Program Institute of Biomedical Sciences Faculty of Medicine University of Chile Santiago Chile; ^2^ Fetal Medicine Unit Clinical Hospital of University of Chile Santiago Chile

**Keywords:** cDC1s, DC activation, IRE1, UPR, XBP1s

## Abstract

The intracellular mechanisms safeguarding DC function are of biomedical interest in several immune‐related diseases. Type 1 conventional DCs (cDC1s) are prominent targets of immunotherapy typified by constitutive activation of the unfolded protein response (UPR) sensor IRE1. Through its RNase domain, IRE1 regulates key processes in cDC1s including survival, ER architecture and function. However, most evidence linking IRE1 RNase with cDC1 biology emerges from mouse studies and it is currently unknown whether human cDC1s also activate the enzyme to preserve cellular homeostasis.

In this work, we report that human cDC1s constitutively activate IRE1 RNase in steady state, which is evidenced by marked expression of IRE1, XBP1s, and target genes, and low levels of mRNA substrates of the IRE1 RNase domain. On a functional level, pharmacological inhibition of the IRE1 RNase domain curtailed IL‐12 and TNF production by cDC1s upon stimulation with TLR agonists. Altogether, this work demonstrates that activation of the IRE1/XBP1s axis is a conserved feature of cDC1s across species and suggests that the UPR sensor may also play a relevant role in the biology of the human lineage.

## Graphical abstract

Human type 1 conventional dendritic cells (cDC1s) display constitutive activation of the IRE1/XBP1s axis of the UPR in steady state. Proinflammatory cytokine production by activated cDC1s is partly dampened upon inhibition of the IRE1 RNase domain, uncovering a role of the pathway in the biology of the human DC lineage.

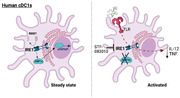

## Introduction

Type 1 conventional DCs (cDC1s) are relevant candidates of immunotherapy due to their superior ability to prime CD8^+^ T cells against tumors and virally infected cells [1, 2, 3, 4, 5]. An intracellular mechanism gaining interest in cDC1 homeostasis is the pathway regulated by the IRE1 (inositol‐requiring enzyme 1, alpha) sensor of the unfolded protein response (UPR), which is an adaptive response aiming to prevent the detrimental effects of ER stress [6, 7]. IRE1 possesses an endoribonuclease (RNase) domain that mediates unconventional splicing of the mRNA coding for the transcription factor XBP1s (X‐box binding protein spliced), master regulator of ER biogenesis [7, 8]. Additionally, the RNase domain of IRE1 cleaves various mRNAs and microRNAs (miRNAs) through a process termed “Regulated IRE1‐Dependent Decay” (RIDD), which alleviates the detrimental effects of ER stress and regulates several processes including inflammation and apoptosis [9, 10].

Notably, the IRE1/XBP1s axis regulates the development of cDCs and plasmacytoid DCs (pDCs) [11] and on a functional level, IRE1 RNase is constitutively active in cDC1s [11, 12]. In this DC subtype, the enzyme controls a core of transcripts involved in ER homeostasis, antigen presentation, and survival [13, 14]. The selectivity of the IRE1/XBP1s axis in cDC1s is underscored in microarray studies of XBP1‐deficient cells, which changed the transcriptomic landscape of cDC1s but not cDC2s [12]. However, despite these findings, most evidence linking IRE1 RNase activity and DC biology emerges from studies in mice models, and little is known about the role of the enzyme in human DC homeostasis. This is a relevant issue since mouse DCs are aligned to human DC homologs [15, 16], although functional differences also exist, particularly in processes related to antigen presentation [17]. Thus, at present, it is unclear whether human cDC1s also co‐opt IRE1 RNase for proper function. In this work, we report that human cDC1s display constitutive activation of the IRE1/XBP1s axis, which contributes to activation upon innate stimulation. Furthermore, our data support the notion that activation of the IRE1/XBP1s axis is a common feature of cDC1s across species.

## Results and discussion

### Human cDC1s activate the IRE1/XBP1s axis in steady state

**Figure 1:**
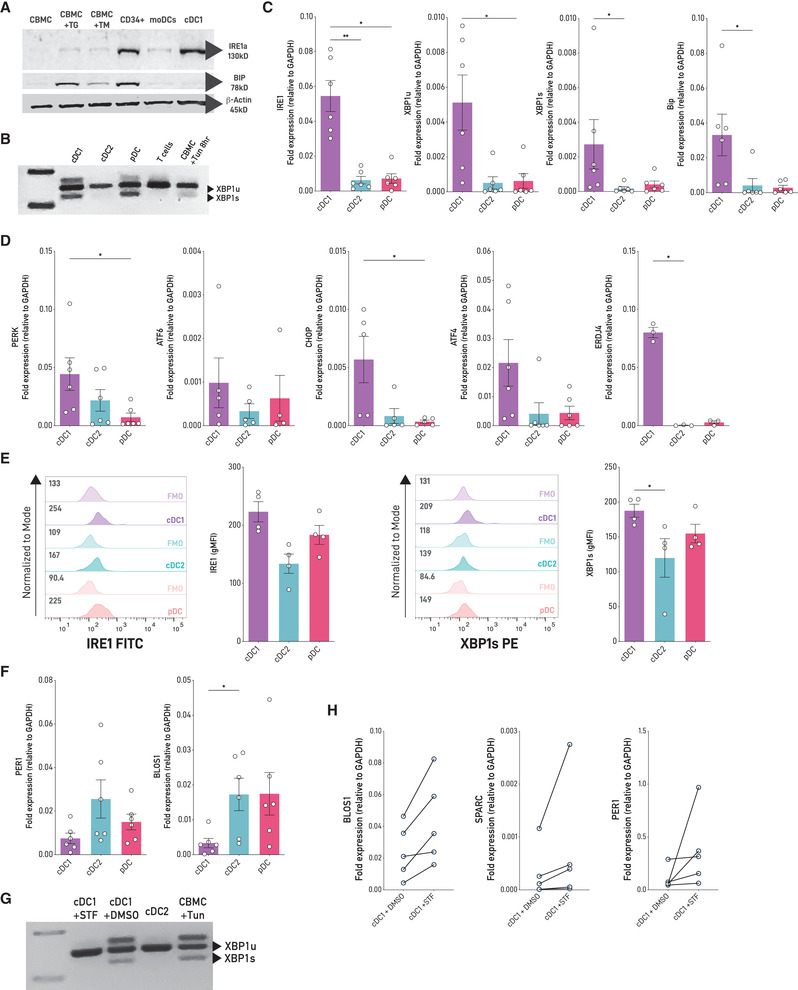
**Human cDC1s co‐opt the IRE1/XBP1s axis in steady state (A)**. Protein levels of IRE1 and BiP were assessed through western blot in OP9‐DL1‐differentiated cDC1s compared to CD34^+^ hematopoietic precursors and monocyte‐derived DCs (moDCs). Cord blood mononuclear cells (CBMC) untreated or treated with tunicamycin (1 μg/mL) or thapsigargin (500 nM) for 8 h were used as negative and positive controls of ER stress‐induced UPR activation. Data are representative of two independent experiments (n = 2). **(B)** In vitro OP9‐DL1‐differentiated cDC1s and cDC2s and cord blood pDCs were identified and isolated using multiparametric flow cytometry and fluorescence activated cell sorting, respectively. *XBP1* splicing was determined using conventional PCR. CBMC treated with tunicamycin and CD3^+^ T cells were used as positive and negative controls, respectively. Data are representative of four independent experiments (n = 4), compared to cDC2s and pDCs. **(C)** mRNA expression of *IRE1*, *XBP1*, *and BiP* relative to *GAPDH* in human DC subsets. Graph shows a pool of six independent experiments (n = 6), in which each dot represents one independent sample. **(D)** mRNA expression of *PERK*, *ATF6* and downstream signaling effectors relative to *GAPDH* in human DC subsets. Graph shows a pool of five independent experiments, in which each dot represents one independent sample (n = 5). **(E)** IRE1 and XBP1s protein expression in DC subsets from cord blood mononuclear cells using flow cytometry. Graphs show a pool of four independent experiments, in which each dot represents one independent sample (n = 4). **(F)** Expression of Regulated IRE1‐dependent decay (RIDD) targets *BLOS1* and *PER1* relative to *GAPDH* was determined by qPCR. Graph shows a pool of five independent experiments in which each dot represents one independent sample (n = 5). **(G)** Conventional PCR of *XBP1 spliced/unspliced* from cDC1s treated with the IRE1 inhibitor STF‐083010 (60 μM, 6 h) or DMSO (vehicle). CBMC treated with tunicamycin were used as positive control. Data are representative of six independent experiments (n = 6). **(H)** Gene expression of RIDD targets *BLOS1*, *PER1*, and *SPARC* was assessed in cDC1s treated with the IRE1 inhibitor STF‐083010 through qPCR. Vehicle‐treated cDC1s were used as control. Graph shows a pool of five independent experiments in which each dot represents one independent sample (n = 5). Error bars in **(C**; **D**; **E**; **F**; and **H)** indicate the mean ± SEM. Statistical test in (**C**; **D**; **E**; **F**; and **H**: Mann‐Whitney nonparametric test ***p < 0.001; **p < 0,01; *p < 0.05).

Human DCs are found in low numbers in circulation, which complicates in‐depth studies and clinical applications. To circumvent this issue, we used a differentiation protocol for culture of human cDCs reported by Kirkling et al. [18] and Balan et al. [19], in which CD34^+^ hematopoietic progenitors cocultured with the Notch ligand‐expressing stromal cell line OP9‐DL1 generate high numbers of *bona‐fide* cDCs (referred to as “OP9‐DL1/DC cultures”; Supporting information Fig. S1). To evaluate if human cDC1s express the UPR sensor IRE1, we determined expression of the protein in cDC1s sorted from OP9‐DL1/DC cultures and compared it to cord blood mononuclear cells (CBMC), CD34^+^ cells, and monocyte‐derived DCs (moDC). Data depicted in Fig. 1A show that human cDC1s express higher levels of IRE1 protein than CBMC and moDCs and similar levels than CD34^+^ cells. Interestingly, human cDC1s express lower levels of BiP than CMBC treated with the pharmacological UPR inducers thapsigargin (TG) or tunicamycin (TM), indicating that expression of IRE1 in human cDC1s is not associated with activation of acute ER stress (Fig. 1A). In fact, cDC1s were highly sensitive to ER stress‐induced cell death, as revealed by a marked reduction in the frequency of the DC subtype in response to TM treatment (Supporting information Fig. S2). Next, we compared expression of canonical UPR members among human DC subsets. To this end, we isolated cDC1s and cDC2s from OP9‐DL1/DC cultures and pDCs from cord blood (Supporting information Fig. S1). Data depicted in Fig. 1B indicate that steady‐state cDC1s express marked levels of *XBP1*s mRNA, feature that was also observed in pDCs, as reported in mice [11]. Interestingly, human cDC1s also expressed higher levels of *IRE1*, *XBP1s*, the XBP1s target *ERDJ4* and *BiP* compared to cDC2 counterparts and higher levels of *IRE1*, *XBP1u* (unspliced)*, PERK*, and *CHOP* mRNA compared to pDCs (Fig. 1C, D). To extend these findings to tissue‐derived DCs, we quantified XBP1s and IRE1 expression by flow cytometry in cDC1s, cDC2s, and pDCs isolated from fresh cord blood samples. We found that cDC1s expressed higher levels of XBP1s than cDC2s and expressed similar levels of IRE1 and XBP1s compared to pDCs (Fig. 1E). Altogether, these results indicate that cDC1s constitutively activate the IRE1/XBP1s axis and endorse potential activation of additional UPR branches.

Mouse cDC1s display constitutive IRE1 RNase activity [12]. We found that two canonical RIDD targets, *BLOS1* and *PER1* mRNA, were expressed at lower levels in human cDC1s compared to cDC2s and pDCs, which is suggestive of RIDD activation (Fig. 1F). To test this hypothesis, we treated sorted cDC1s with STF‐083010, a small IRE1 inhibitor that blocks IRE1 RNase activity without affecting its kinase activity [20]. STF‐083010 treatment efficiently inhibited XBP1 splicing in cDC1s (Fig. 1G) and regulated RIDD activity, as revealed by a trend towards an increased expression of *BLOS1*, *PER1*, and *SPARC* mRNAs (Fig. 1H). Taken together, these results show that human cDC1s constitutively activate the IRE1/XBP1s axis and show signs of RIDD induction in steady state.

### IRE1 RNase blockade prevents cytokine production by cDC1s upon TLR triggering

**Figure 2:**
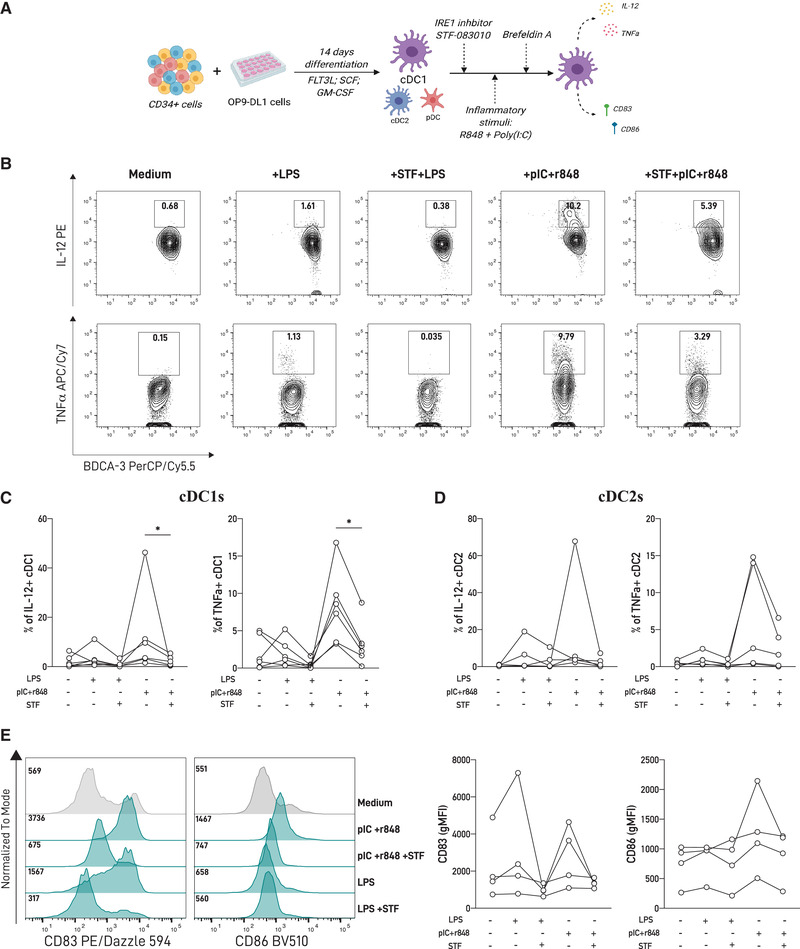
**Activation of the IRE1/XBP1s axis by cDC1s modulates innate responses. (A)** Experimental scheme of cDC1 activation with toll‐like receptor agonists in presence of an IRE1 RNase (STF‐083010) inhibitor. **(B, C)** cDC1s differentiated from OP9‐DL1/DC cultures were treated for 2 h with the IRE1 inhibitor STF‐083010 (60 μM) prior to 16 h stimulation with LPS (1 μg/mL) or R848 (5 μg/mL) and poly(I:C) (5 μg/mL); and IL‐12 and TNF expression was determined using flow cytometry. Flow cytometry plots are representative of six independent experiments (n = 6) and graphs show a pool of six independent experiments in which each dot represents one independent sample (n = 6). **(D)** IL‐12 and TNF expression was also determined by flow cytometry in cDC2s from the OP9‐DL1/DC cultures treated with STF‐083010 prior to LPS and poly(I:C) stimulation. Graphs show a pool of five independent experiments, in which each dot represents one independent sample (n = 5). **(E)** CD83 and CD86 expression in cDC1 treated with R848 and poly(I:C) with or without IRE1 inhibition with STF‐083010. Histograms are representative of four independent experiments (n = 4) and graphs show a pool of four independent experiments in which each dot represents one independent sample (n = 4). Error bars in (**C, D,** and **E**) indicate the mean ± SEM. Statistical test used in (**C‐E**) was Wilcoxon matched‐pairs signed rank ***p < 0.001; **p < 0,01; *p < 0.05.

In myeloid cells, a direct connection between the IRE1/XBP1s axis and innate recognition is described [7, 21, 22]. Macrophages require XBP1s for optimal production of inflammatory cytokines downstream of TLR signaling [21, 23]. Similarly, the production of IL‐12/23, TGF‐β, and IL‐1β by human moDCs is partly dependent of the IRE1/XBP1 axis [22, 24], and blockade of IRE1 RNase in mouse cDC1 equivalents reduced IL‐12 production upon activation with tumor cell lysates [25]. To examine whether basal IRE1 RNase activity in human cDC1s has a functional role, we measured the production of proinflammatory cytokines and expression of costimulatory molecules by cDC1s upon innate stimulation, in the presence or absence of STF‐083010 (scheme depicted in Fig. 2A). Stimulation with a variety of TLR agonists revealed that the combination of the TLR3 agonist polyinosinic:polycytidylic acid (poly[I:C]) and the TLR7/8 agonist R848 resulted in robust production of TNF and IL‐12, and upregulation of CD83 and CD86 by cDC1s, as reported [18, 19] (Supporting information Fig. S3 and Fig. 2A‐C). Treatment with STF‐083010 prior to TLR triggering significantly decreased IL‐12 and TNF production in cDC1s compared to TLR‐activated counterparts in presence of control vehicle (Fig. 2B, C). Interestingly, inhibition of IRE1 RNase activity did not result in significant reduction of cytokine production by cDC2s (Fig. 2D and Supporting information Fig. S4), indicating that the effect of IRE1 blockade upon TLR activation is manifested in the cDC1 lineage. Regarding expression of costimulatory molecules, we observed that IRE1 RNase blockade prior to TLR triggering did not change CD83 or CD86 expression by cDC1s (Fig. 2E). Finally, we also investigated if acute ER stress could lead to spontaneous cytokine production by human cDC1s (Supporting information Fig . S5). We found that TM treatment does not elicit IL‐12 production by these cells, indicating that UPR activation in absence of TLR triggering is not sufficient to induce cytokine production by human cDCs. Altogether, these data suggest that pharmacological inhibition of the IRE1 RNase domain dampens aspects of cDC1 activation upon TLR triggering.

### Concluding remarks

The UPR is a complex network of signaling pathways sensing perturbations that affect ER function which extend beyond its canonical role. In this context, the “physiological UPR” plays an important role in the homeostasis of several immune cells including DCs [12, 14]. The findings presented here confirm that human cDC1s, like their mouse counterparts, co‐opt the IRE1/XBP1s pathway in steady state. To our knowledge, this is the first study reporting an interplay between UPR components and human cDC1 biology. Our results also show basal *XBP1s* expression in pDCs, which is in line with evidence reported in mice [11]. In addition, data presented here suggest that human cDC1s display basal RIDD activity. These results are interesting considering that RIDD has emerged as a critical regulator of inflammation and apoptosis [9], although its role in human immunity remains conspicuously studied. However, future work is required to formally demonstrate if RIDD regulates functional features of human cDC1 in physiology, such as survival or antigen cross‐presentation, which are parameters observed in mouse cDC1s upon enforced activation of RIDD [12, 14]. Furthermore, an additional question emerging from these findings is to interrogate if reported regulators of IRE1 activity that also control cDC function in mice models, such as mTOR signaling [26, 27], can cross‐regulate the IRE1/XBP1s axis in human cDCs.

On a functional level, pharmacological blockade of IRE1 RNase upon TLR triggering in cDC1s curtails archetypical parameters of DC activation, which include IL‐12 and TNF production. These data differ from previous studies in moDCs where XBP1s blockade did not affect LPS‐induced TNF production [24, 28], supporting the notion that IRE1 activity may operate differently among DC subsets, as previously reported in mice [12]. In this context, additional approaches, such as genetic deletions of IRE1 RNase and XBP1s in human cDCs will consolidate the observations generated in this work. Along these lines, the advent of recent methods for cDC culture or expansion have been highly valuable for the study of molecular mechanisms safeguarding human cDC function, and conceivably, optimized protocols for genetic manipulation of these cells will be developed in the short term. Overall, the findings presented in this work demonstrate that activation of IRE1 RNase is a common feature of the cDC1 subset across species and highlight that the IRE1/XBP1s axis operates as a strict regulatory circuit in human cDC1s contributing to aspects such as cytokine production. Furthermore, this work contributes to pave the road for future studies regulating IRE1 RNase and XBP1s to fine tune human DC function in biomedical settings.

## Material and methods

### Human samples

Cord blood was collected after birth, immediately after umbilical cord section, from informed and consenting mothers at the time of elective cesarean section at full term pregnancy. All participating individuals were required to understand the study and sign with informed consent.

### OP9/DL1 Cell line

OP9 cells expressing Notch ligand DL1 (OP9‐DL1) were kindly donated by Dr. Juan Carlos Zuñiga‐Pflucker from Sunnybrook Research Institute, Toronto, Canada. OP9‐DL1 cells were cultured in MEM—a medium supplemented with 20% FCS, 1% penicillin/streptomycin (Gibco), and 1 mM sodium pyruvate (Gibco) at 37°C, 5% CO_2_, 24 h prior use, cells were treated with mitomycin C at 10 μg/mL for 2 h, harvested, washed with PBS, resuspended in OP9 medium, and cultured at a 96‐well U‐bottom plate (5000/well).

### Cord blood cell isolation

CBMCs were isolated using density centrifugation. Cord blood samples were diluted in Dulbecco's phosphate‐buffer saline (DPBS 1×, Gibco) 1:1 prior to addition of Ficoll‐Paque Plus (GE Healthcare) and centrifugation at 1200 *g*, at room temperature for 25 min to allow layer separation. After recovery of the mononuclear cell layer, cells were washed twice with DBPS and then treated with ACK lysing buffer 1× (Gibco) for 10 min. Cells were then washed with DPBS 1×, and frozen and stored at −80°C until further use or resuspended in DPBS with 1% fetal calf serum and 0.1% EDTA for CD34+ enrichment using the CD34 MicroBead UltraPure Kit (Miltenyi) or stained for cell sorting. For ER stress‐induced UPR positive controls, CBMC samples were treated for 8 h with TM (1 μg/mL) or TG (500 nM) prior to RNA or protein extraction. CBMC cultured in medium was often used as a negative control.

### In vitro generation of cDC1 dendritic cells

Notch‐mediated differentiation of cDC1s was performed based on the protocols described by Kirkling et al. [18] and Balan et al. [19] Purified CD34+ hematopoietic progenitors were cultured (3000/well) in 96‐well U‐bottom plates with preseeded OP9‐DL1 stromal cells (5000/well) in MEM—a medium supplemented with 10% FCS (Gibco) 1% penicillin or streptomycin (Gibco), 20 ng/mL GM‐CSF (R&D Systems), 20 ng/mL SCF (R&D Systems) and 200 ng/mL FLT3‐ligand (FLT3‐L, R&D Systems) for 14 days in a 5% CO_2_ incubator, 37°C. Half the volume of media and cytokines was replaced at day 7. At the end of culture, cells were harvested, cell numbers determined using trypan blue and stained for flow cytometric analysis or resuspended in culture media for further functional analysis.

### Flow cytometry

Flow cytometry and cell sorting protocols were performed according to the guidelines for the use of flow cytometry and cell sorting in immunological studies [29]. Isolated CBMC or cultured cDC1 cell suspensions were stained for multicolor analysis using fluorochrome‐conjugated antibodies for DC progenitor and DC subsets identification detailed in Supporting information Table S1. Cell samples were resuspended in PBS 1× for viability dye staining with either Zombie UV or Zombie Aqua Fixable Viability Kit (Biolegend) and incubated at room temperature in the dark for 20 min. Cells were then washed with PBS, resuspended in FACS buffer (PBS, 1% FCS, 0.1% EDTA) and incubated with the corresponding antibodies, previously diluted in FACS buffer, for 30 min at 4°C in the dark. For functional analyses, intracellular cytokine staining was performed for 30 min in the dark at 4°C in cultured DC samples after surface staining, fixation, and permeabilization (Biolegend). Acquisition and analysis were performed on a LSR Fortessa X‐20 running FACSDIVA software and subsequent data analysis was performed with FlowJo software X (FlowJo, LLC).

### Cell sorting

Isolated CBMC or cDC1 cell suspensions were resuspended in FACS buffer and stained using fluorochrome‐conjugated antibodies for DC progenitor cell sorting detailed in Supporting information Table S1. Stained cell suspension was filtered by a 30 μm nylon mesh and sorted by flow cytometry. Dead cells were excluded using a viability dye staining with Zombie NIR Fixable Viability Kit (Biolegend). Sorting was performed with a FACS Aria III (BD Biosciences) and cells were sorted into 350 μL of FCS to be used for functional analysis and protein isolation, or 350 μL of RLB Buffer from the Rneasy Plus Micro Kit (Qiagen) for RNA isolation.

### Dendritic cell functional analysis

For cytokine production, DCs differentiated from OP9‐DL1/DC cultures were replated in 96‐well U‐bottom plates in 200 μL of culture media (2,00,000 cells/well) and stimulated with R848 (5 μg/mL, Invivogen), poly(I:C) (10 μg/mL, Invivogen), LPS (5 ng/mL, Sigma), and/or CpG (ODN 2216, 5 μg/mL, Invivogen) for 16 h. Brefeldin A (10 μg/mL, Sigma) was added after 5 h. For UPR inhibition experiments, STF‐083010 (60 μM, Sigma) was added 2 h prior to TLR‐agonists stimulation in half the culture media.

### Inhibitor experiments

For IRE1 Rnase inhibition experiments, OP9‐differentiated cDC1 cells were sorted and replated in 96‐well U‐bottom plates in 200 μL of culture media (200,000 cells/well) and treated for 6 h with the IRE1 Rnase small inhibitors STF‐083010 (60 μM, Sigma) prior to RNA isolation.

### RNA isolation, cDNA generation, and PCR/qPCR analysis

RNA isolation was performed using the Rneasy Plus Micro Kit (Qiagen). Sorted cells were immediately resuspended in the lysis buffer RLT (Qiagen) and RNA isolation procedure was done according to manufacturer's instructions. Integrity and concentration of RNA was assessed using a NanoDrop Lite (Thermo Scientific). cDNA was obtained using the M‐MLV Reverse Transcriptase Kit (Invitrogen) following manufacturer's guidelines and SYBR green‐based qPCR was performed using LightCycler 480 System (Roche). XBP1 splicing analysis was done by conventional PCR. The pharmacologic ER‐stress inducer TM was used as a positive control for UPR‐related genes expression induction. Primers used for PCR and qPCR are detailed in Supporting information Table S2.

### Protein extraction and western blot

For protein isolation, sorted cDC1s were washed twice with ice‐cold PBS at 500 *g* for 10 min after which pelleted cells were resuspended in 50 μL of E1A buffer (1% NP40, 20 mM HEPES, pH 7.9, 250 mM NaCl, and 1 mM EDTA complemented with Complete‐ULTRA [Roche] and PhosSTOP [Roche]). Samples were then incubated on ice for 15 min, vortexed every 5 min, then spin at 12,000 *g* to remove insoluble material, and stored at −80°C until use. Prior to SDS–PAGE, samples were resuspended in loading dye and heated at 95°C for 10 min. After wet transfer to polyvinyldifluoride membrane (Immobilon; Millipore), proteins were analyzed by immunoblotting and visualized by chemiluminescence (SuperSignalTM West Pico Chemiluminescent Substrate, Thermo Fisher Scientific). Primary antibodies used were IRE1‐α Rabbit mAb (clone 14C10; 1/1000), BiP Rabbit mAb (clone C50B12; 1/3000), and β‐actin mouse mAb (clone 8H10D10; 1/5000), and secondary antibodies were anti‐rabbit‐ HRP (#7074, 1/4000) and anti‐mouse‐HRP (#7076; used 1/4000), all purchased from Cell signaling.

### Statistical analysis

Statistical analyses were performed using Mann‐Whitney tests for DC subsets comparison and paired Wicoxon signed rank test for cytokine and surface marker analysis in cDC1 assays. Results with a *p*‐value of 0.05 or less were considered significant. Mean values, SD, and statistics were calculated using Graphpad Prism Software 8. No criteria of inclusion of exclusion of data were used in this study.

## Conflict of interest

The authors declare no commercial or financial conflict of interest.

## Author contributions

PGG and FO worked on study design and conceptualization. MP and DG worked on sample handling and collection. PGG and DF processed samples, performed experiments, and analyzed data. FO supervised the project. PGG and FO interpreted data and wrote the manuscript with input from all coauthors. All authors reviewed the final draft.

## Ethics approval statement

This study was performed with compliance with the Declaration of Helsinki. The study was approved by the Ethics Committee for Research in Human Beings from the Faculty of Medicine at University of Chile and the Scientific Ethics Committee from Clinical Hospital of University of Chile.

## Patient consent statement

Written informed consent was obtained from all participants prior to recruitment.

### Peer review

The peer review history for this article is available at https://publons.com/publon/10.1002/eji.202149774

**Abbreviations**
CBMC: cord blood mononuclear cells
cDC1s: conventional DCs
FLT3‐L: FLT3‐ligand
IRE: inositol‐requiring enzyme 1 alpha
miRNAs: microRNAs
moDC: monocyte‐derived DCs
pDCs: plasmacytoid DCs
RIDD: Regulated IRE1‐Dependent Decay
TG: thapsigargin
TM: tunicamycin
UPR: unfolded protein response
XBP1s: X‐box binding protein

## Supporting information

Supporting Information

## Data Availability

The data that support the findings of this study are available from the corresponding author upon reasonable request.

## References

[1] Collin, M. and Bigley, V. , Human dendritic cell subsets: an update. Immunology. 2018. 154: 3–20. 29313948 10.1111/imm.12888 PMC5904714

[2] Audsley, K. M. , McDonnell, A. M. and Waithman, J. , Cross‐presenting XCR1+ dendritic cells as targets for cancer immunotherapy. Cells 2020. 9: 565. 10.3390/cells9030565 PMC7140519 32121071

[3] Ashour, D. E. D. , Arampatzi, P. , Pavlovic, V. , Förstner, K. U. , Kaisho, T. , Beilhack, A. , Erhard, F. et al., IL‐12 from endogenous cDC1, and not vaccine DC, is required for Th1 induction. JCI Insight 2020. 5: e135143. 10.1172/jci.insight.135143 PMC7259537 32434994

[4] Mittal, D. , Vijayan, D. , Putz, E. M. , Aguilera, A. R. , Markey, K. A. , Straube, J. , Kazakoff, S. et al., Interleukin‐12 from CD103+ Batf3‐dependent dendritic cells required for NK‐cell suppression of metastasis. Cancer Immunol. Res. 2017. 5: 1098–1108. 29070650 10.1158/2326-6066.CIR-17-0341

[5] Ghilas, S. , Ambrosini, M. , Cancel, J.‐C. , Brousse, C. , Massé, M. , Lelouard, H. , Marc, D. et al., Natural killer cells and dendritic epidermal γδ T cells orchestrate type 1 conventional DC spatiotemporal repositioning toward CD8+ T cells. iScience 2021. 24: 103059. 34568787 10.1016/j.isci.2021.103059 PMC8449251

[6] Hetz, C. , Zhang, K. and Kaufman, R. J. , Mechanisms, regulation and functions of the unfolded protein response. Nat. Rev. Mol. Cell Biol. 2020. 21:421‐438. 32457508 10.1038/s41580-020-0250-z PMC8867924

[7] Grootjans, J. , Kaser, A. , Kaufman, R. J. and Blumberg, R. S. , The unfolded protein response in immunity and inflammation. Nat. Rev. Immunol. 2016. 16: 469–484. 27346803 10.1038/nri.2016.62 PMC5310224

[8] Glimcher, L. H. , Lee, A. H. and Iwakoshi, N. N. , XBP‐1 and the unfolded protein response (UPR). Nat. Immunol. 2020. 21: 963–965. 32616861 10.1038/s41590-020-0708-3

[9] Maurel, M. , Chevet, E. , Tavernier, J. and Gerlo, S. , Getting RIDD of RNA: IRE1 in cell fate regulation. Trends Biochem. Sci. 2014. 39: 245–254. 24657016 10.1016/j.tibs.2014.02.008

[10] Hollien, J. and Weissman, J. S. , Decay of endoplasmic reticulum‐localized mRNAs during the unfolded protein response. Science (80‐) 2006. 313: 104–107. 10.1126/science.1129631 16825573

[11] Iwakoshi, N. N. , Pypaert, M. and Glimcher, L. H. , The transcription factor XBP‐1 is essential for the development and survival of dendritic cells. J. Exp. Med. 2007. 204: 2267–2275. 17875675 10.1084/jem.20070525 PMC2118458

[12] Osorio, F. , Tavernier, S. J. , Hoffmann, E. , Saeys, Y. , Martens, L. , Vetters, J. , Delrue, I. et al., The unfolded‐protein‐response sensor IRE‐1α regulates the function of CD8α + dendritic cells. Nat. Immunol. 2014. 15: 248–257. 24441789 10.1038/ni.2808

[13] Osorio, F. , Lambrecht, B. N. and Janssens, S. , Antigen presentation unfolded: identifying convergence points between the UPR and antigen presentation pathways. Curr. Opin. Immunol. 2018. 52: 100–107. 29754111 10.1016/j.coi.2018.04.020

[14] Tavernier, S. J. , Osorio, F. , Vandersarren, L. , Vetters, J. , Vanlangenakker, N. , Van Isterdael, G. , Vergote, K. et al., Regulated IRE1‐dependent mRNA decay sets the threshold for dendritic cell survival. Nat. Cell Biol. 2017. 19: 698–710. 28459443 10.1038/ncb3518 PMC5563826

[15] Brown, C. C. , Gudjonson, H. , Pritykin, Y. , Deep, D. , Lavallée, V. P. , Mendoza, A. , Fromme, R. et al., Transcriptional basis of mouse and human dendritic cell heterogeneity. Cell 2019. 179: 846–863. 31668803 10.1016/j.cell.2019.09.035 PMC6838684

[16] Guilliams, M. , Dutertre, C. A. , Scott, C. L. , McGovern, N. , Sichien, D. , Chakarov, S. , Van Gassen, S. et al., Unsupervised high‐dimensional analysis aligns dendritic cells across tissues and species. Immunity 2016. 45: 669–684. 27637149 10.1016/j.immuni.2016.08.015 PMC5040826

[17] Segura, E. , Durand, M. and Amigorena, S. , Similar antigen cross‐presentation capacity and phagocytic functions in all freshly isolated human lymphoid organ‐resident dendritic cells. J. Exp. Med. 2013. 210: 1035–1047. 23569327 10.1084/jem.20121103 PMC3646495

[18] Kirkling, M. E. , Cytlak, U. , Lau, C. M. , Lewis, K. L. , Resteu, A. , Khodadadi‐Jamayran, A. , Siebel, C. W. et al., Notch signaling facilitates in vitro generation of cross‐presenting classical dendritic cells. Cell Rep. 2018. 23: 3658–3672. 29925006 10.1016/j.celrep.2018.05.068 PMC6063084

[19] Balan, S. , Arnold‐Schrauf, C. , Abbas, A. , Couespel, N. , Savoret, J. , Imperatore, F. , Villani, A. ‐C. et al., Large‐scale human dendritic cell differentiation revealing notch‐dependent lineage bifurcation and heterogeneity. Cell Rep. 2018. 24: 1902–1915. 30110645 10.1016/j.celrep.2018.07.033 PMC6113934

[20] Papandreou, I. , Denko, N. C. , Olson, M. , Van Melckebeke, H. , Lust, S. , Tam, A. , Solow‐Cordero, D. E. et al., Identification of an Ire1alpha endonuclease specific inhibitor with cytotoxic activity against human multiple myeloma. Blood 2011. 117: 1311–1314. 21081713 10.1182/blood-2010-08-303099 PMC3056474

[21] Martinon, F. , Chen, X. , Lee, A. H. and Glimcher, L. H. , TLR activation of the transcription factor XBP1 regulates innate immune responses in macrophages. Nat. Immunol. 2010. 11: 411–418. 20351694 10.1038/ni.1857 PMC3113706

[22] Márquez, S. , Fernández, J. J. , Terán‐Cabanillas, E. , Herrero, C. , Alonso, S. , Azogil, A. , Montero, O. et al., Endoplasmic reticulum stress sensor IRE1α enhances IL‐23 expression by human dendritic cells. Front. Immunol. 2017. 8: 639. 28674530 10.3389/fimmu.2017.00639 PMC5475432

[23] Zhang, L. , Pavicic, P. G. , Datta, S. , Song, Q. , Xu, X. , Wei, W. , Su, F. et al., Unfolded protein response differentially regulates TLR4‐induced cytokine expression in distinct macrophage populations. Front. Immunol. 2019. 10: 1–12. 31293572 10.3389/fimmu.2019.01390 PMC6598306

[24] Betts, B. C. , Locke, F. L. , Sagatys, E. M. , Pidala, J. , Walton, K. , Menges, M. , Reff, J. et al., Inhibition of human dendritic cell er stress response reduces t cell alloreactivity yet spares donor anti‐tumor immunity. Front. Immunol. 2018. 9: 2887. 30574153 10.3389/fimmu.2018.02887 PMC6291501

[25] Medel, B. , Costoya, C. , Fernandez, D. , Pereda, C. , Lladser, A. , Sauma, D. , Pacheco, R. et al., IRE1α activation in bone marrow‐derived dendritic cells modulates innate recognition of melanoma cells and favors CD8+ T cell priming. Front. Immunol. 2019. 9:3050. 30687308 10.3389/fimmu.2018.03050 PMC6338037

[26] Benhamron, S. , Pattanayak, S. P. , Berger, M. and Tirosh, B. , mTOR activation promotes plasma cell differentiation and bypasses XBP‐1 for immunoglobulin secretion. Mol. Cell. Biol. 2015. 35: 153–166. 25332234 10.1128/MCB.01187-14 PMC4295374

[27] Sathaliyawala, T. , O'Gorman, W. E. , Greter, M. , Bogunovic, M. , Konjufca, V. , Hou, Z. E. , Nolan, G. P. et al., Mammalian target of rapamycin controls dendritic cell development downstream of Flt3 ligand signaling. Immunity 2010. 33: 597–606. 20933441 10.1016/j.immuni.2010.09.012 PMC2966531

[28] Riaz, T. A. , Junjappa, R. P. , Handigund, M. , Ferdous, J. , Kim, H. R. and HJ, C. , Role of endoplasmic reticulum stress sensor IRE1α in cellular physiology, calcium, ros signaling, and metaflammation. Cells. 2020. 9:1160. 10.3390/cells9051160 PMC7290600 32397116

[29] Cossarizza, A. , Chang, H. D. , Radbruch, A. , Abrignani, S. , Addo, R. , Akdis, M. , Andrä, I. et al., Guidelines for the use of flow cytometry and cell sorting in immunological studies (third edition). Eur. J. Immunol. 2021. 51: 2708–3145. 34910301 10.1002/eji.202170126 PMC11115438

