# Supplementary material for: Human cDC1s display constitutive activation of the UPR sensor IRE1
Source: Eur J Immunol. 2022 Apr 22;52(7):1069–76. doi: 10.1002/eji.202149774 (PMC9541385; doi:10.1002/eji.202149774)

Supplementary Tables

Table S1. List of antibodies used for flow cytometry and cell sorting.

Panel 1. DC subsets and DC phenotype				
**Antibody**	**Clone**	**Source**	**Identifier**	**Dilution**
Anti-human IRE1 FITC	B-12	Santa Cruz	sc-390960	1/50
Anti-human XBP1s PE	143F	Biolegend	647503	1/50
Anti-human IL-12/IL-23p40 PE	C11.5	Biolegend	501806	1/50
Anti-human CD83 PE/Dazzle 594	HB15e	Biolegend	305327	1/100
Anti-human CD141 PerCP/Cy5.5	M80	Biolegend	344111	1/100
Anti-human CD1c PE/Cy7	L161	Biolegend	331515	1/100
Anti-human CLEC9A (DNGR-1) APC	8F9	Biolegend	353805	1/100
Anti-human SIRP1a (CD172a) Alexa Fluor 700	602411	R&D Systems	FAB4546N	1/100
Anti-human TNFα APC/Cy7	MAb11	Biolegend	502943	1/50
HLA-ABC APC/Fire 750	W6/32	Biolegend	311443	1/200
CD11c BV421	3.9	Biolegend	301627	1/100
Anti-human CD86 BV510	IT2.2	Biolegend	305431	1/100
Anti-human Lineage (CD3, CD14, CD16, CD19, CD20, CD56) BV510	OKT3; M5E2; 3G8; HIB19; 2H7; HCD56	Biolegend	348807	1/200
Anti-human CD45 BV605	2D1	Biolegend	368524	1/200
Anti-human HLA-DR BV785	L243	Biolegend	307641	1/200
Anti-human CD11c BUV396	B-ly6	BD Biosciences	563788	1/100
Zombie UV fixable viability kit	-	Biolegend	423108	1/1000
**Panel 2. Sorting of DC subsets**				
**Antibody**	**Clone**	**Source**	**Identifier**	**Dilution**
Anti-human CD11c FITC	3.9	Biolegend	301603	1/100
Anti-human CD123 PE	7G3	BD Biosciences	554529	1/100
Anti-human HLA-DR PE/Dazzle 594	L243	Biolegend	307653	1/200
Anti-human CD141 PerCP/Cy5.5	M80	Biolegend	344111	1/100
Anti-human CD1c PE/Cy7	L161	Biolegend	331515	1/100
Anti-human CLEC9A (DNGR-1) APC	8F9	Biolegend	353805	1/100
Anti-human SIRP1a (CD172a) Alexa Fluor 700	602411	R&D Systems	FAB4546N	1/100
Zombie NIR Fixable Viability Kit	-	Biolegend	423106	1/1000

Table S2. List of primers used for qPCR.

qPCR		
**Gene name**	**Forward (5'-3')**	**Reverse (5'-3')**
hIRE1	TGCTTAAGGACATGGCTACCATCA	CTGGAACTGCTGGTGCTGGA
hXBP-1u	GGCATCCTGGCTTGCCTCCA	GCCCCCTCAGCAGGTGTTCC
hXBP-1s	CGCTTGGGGATGGATGCCCTG	CCTGCACCTGCTGCGGACT
hBLOC1S1	CCCAATTTGCCAAGCAGACA	CATCCCCAATTTCCTTGAGTGC
hPER1	TATACCCTGGAGGAGCTGGA	AGGAAGGAGACAGCCACTGA
hSPARC	GGCCTGGATCTTCTTTCTCC	CCACCACCTCTGTCTCATCA
hPERK	AATGCCTGGGACGTGGTGGC	TGGTGGTGCTTCGAGCCAGG
hCHOP	GGAGCATCAGTCCCCCACTT	TGTGGGATTGAGGGTCACATC
hATF4	GCTAAGGCGGGCTCCTCCGA	ACCCAACAGGGCATCCAAGTCG
hBiP	TGACATTGAAGACTTCAAAGCT	CTGCTGTATCCTCTTCACCAGT
hATF6	ATGAAGTTGTGTCAGAGAACC	CTCTTTAGCAGAAAATCCTAG
hGAPDH	AAGGTGAAGGTCGGAGTCAA	CATGGGTGGAATCATAATGG
**PCR**		
**Gene name**	**Forward (5'-3')**	**Reverse (5'-3')**
hXBP1s/u	CCTGGTTGCTGAAGAGGAGG	CCATGGGGAGATGTTCTGGAG
hACTB	GCGAGAAGATGACCCAGATC	CCAGTGGTACGGCCAGAGG

Figure S1. Identification and sorting strategy of human DCs in cord blood and OP9/DL1-DC cultures.
A. Notch-mediated differentiation of human cDC1s. To promote cDC1 differentiation from hematopoietic precursors, CD34+ cells isolated from progenitors were cultured with pre-seeded OP9-DL1 stromal cells in the presence of FLT3-L (100ug/ml), SCF (20ug/ml) and GM-CSF (20ug/ml)for 14 days in a 5% CO_2 incubator, 37°C. Half the volume of media and cytokines was replaced at day 7. **B.** Sorting strategy of cDC1 and cDC2 cells from OP9/DL1-DC cultures on day 14. pDCs were directly isolated from cord blood samples on Day 1 (**1C**).

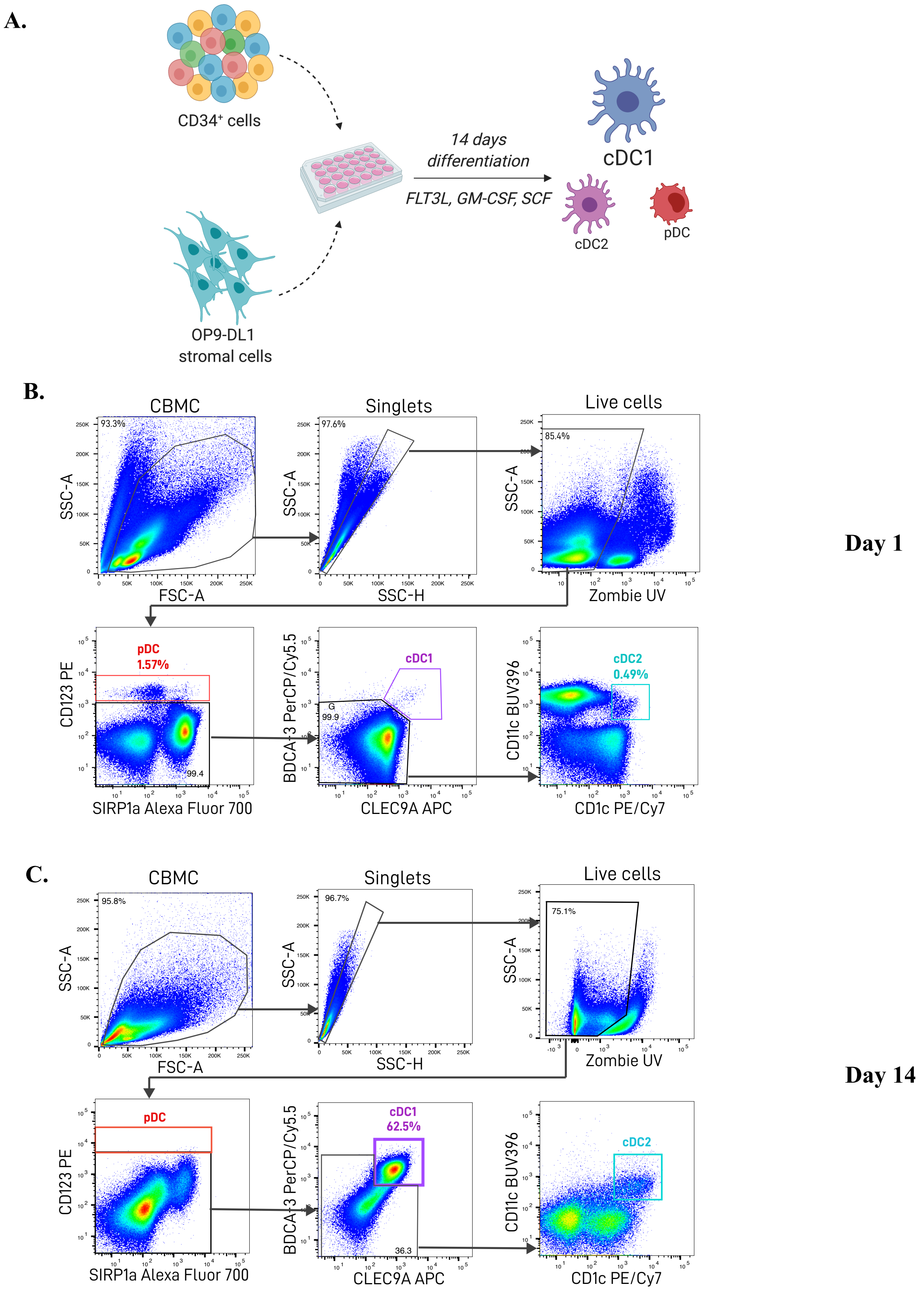

Figure S2. Survival of human cDC under ER stress. *In vitro* OP9-DL1-differentiated dendritic cells were treated with 0,5 or 1 µg/mL Tunicamycin (TM) for 16 hours. Survival of cDC1 (CLEC9A+CD1c-) and cDC2 (CLEC9A-CD1c+) cells was determined by flow cytometry. FACS plots are representative of 3 samples from independent experiments (n=3). The graph shows a pool of 3 independent experiments, all performed under the same experimental conditions, in which each dot represents one independent sample (n=3). Error bars indicate mean ±SEM. Friedman nonparametric test *p<0.05.

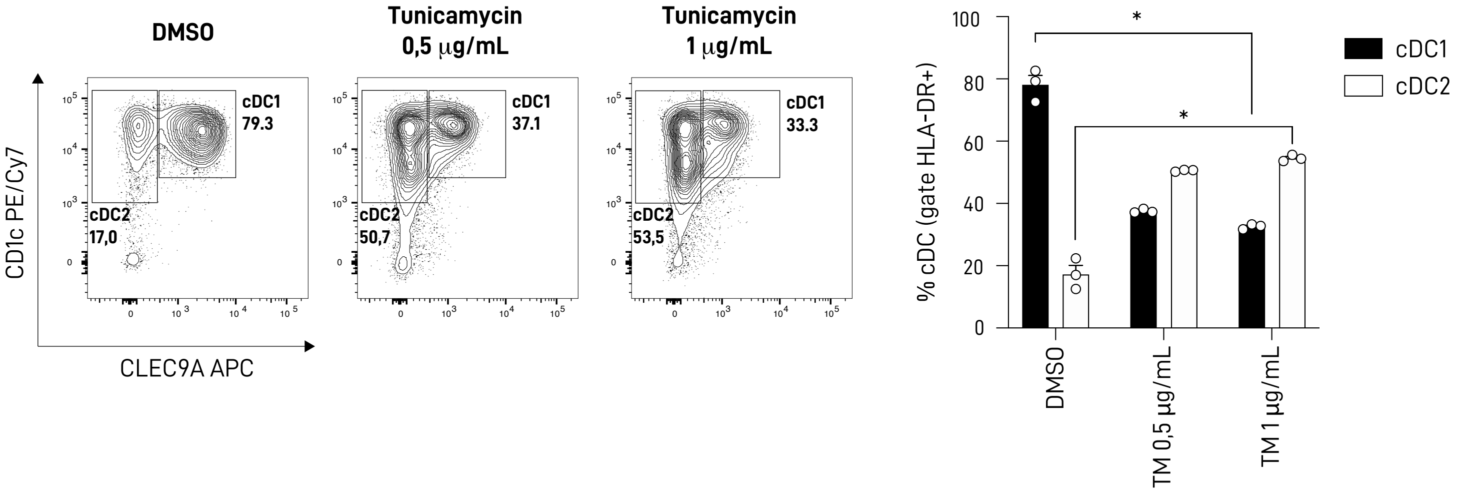

Figure S3. TLR activation of human cDC1s. OP9-differentiated DCs were replated in 96 well U-bottom plates in 200ul of culture media (200.000 cells/well) and stimulated with R848 (5ug/ml, Invivogen) polyinosinic:polycytidylic acid (poly(I:C), 10ug/ml, Invivogen), Lipopolysaccharide (LPS, 5ng/ml, Sigma) and/or CpG (ODN 2216, 5ug/ml, Invivogen) for 16 hours. Brefeldin A (Sigma) was added at a 10ug/ml concentration after 5 hr. **A.** IL-12 and TNFa expression was determined through intracellular flow cytometry staining of cDC1s. FACS plots are representative of 2 different samples (n=2) from independent experiments, all performed under the same conditions. **B.** Expression levels of CD83 and CD86 were also determined in the same 2 samples using flow cytometry.

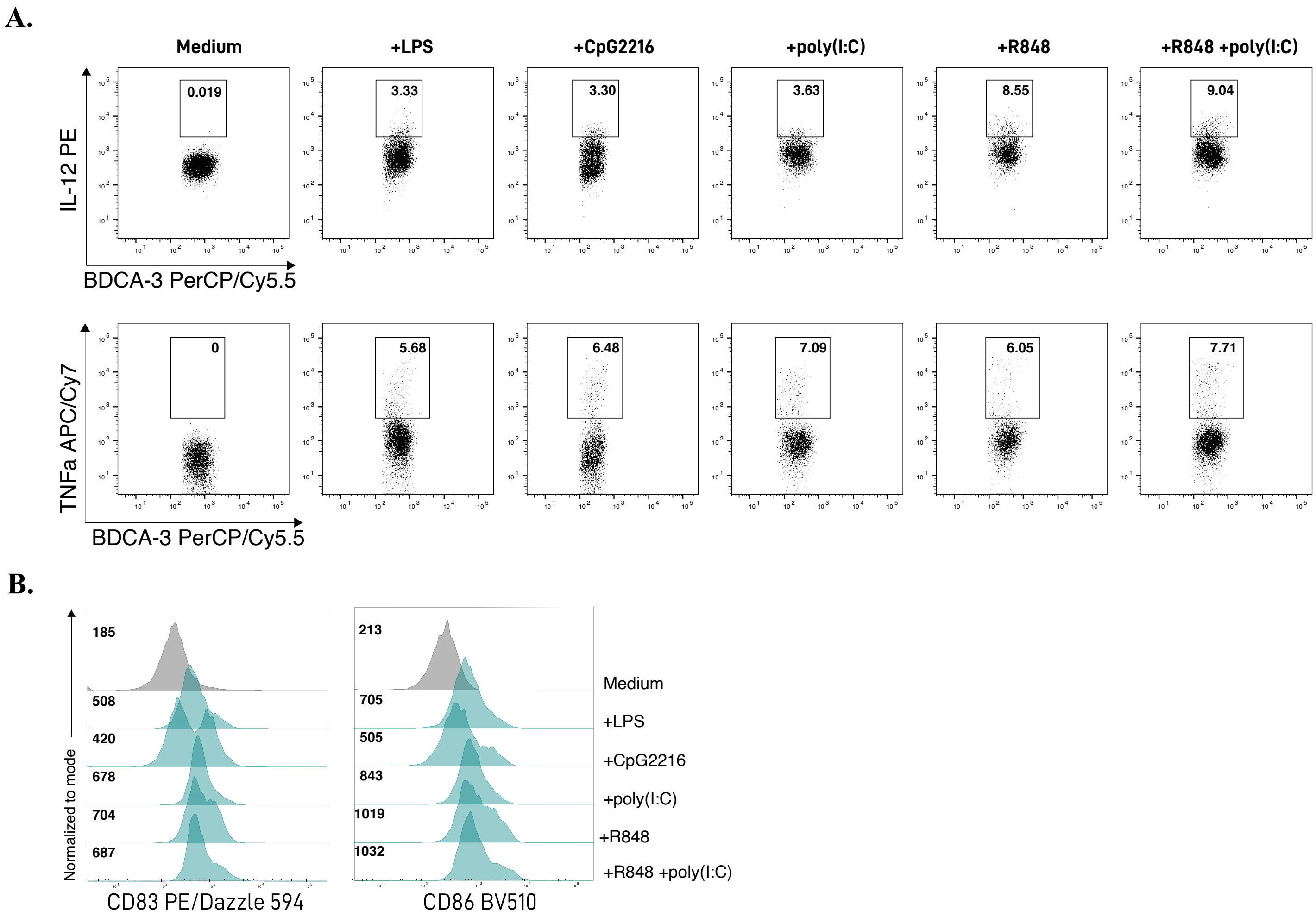

Figure S4. TLR activation of human cDC2s under IRE1 inhibition. IL-12 and TNFa expression was assessed in cDC2s from OP9-DL1/DC cultures that were stimulated with R848 (5ug/ml, Invivogen) and poly-inosinic:polycytidylic acid (poly(I:C), 10ug/ml, Invivogen) or Lipopolysaccharide (LPS, 5ng/ml, Sigma) alone for 16 hours. Brefeldin A (Sigma) was added at a 10ug/ml concentration after 5 hr and cytokine expression was determined using intracellular flow cytometry of cDC2 cells. FACS plots are representative of 5 different samples (n=5) from independent experiments, all performed under the same experimental conditions.

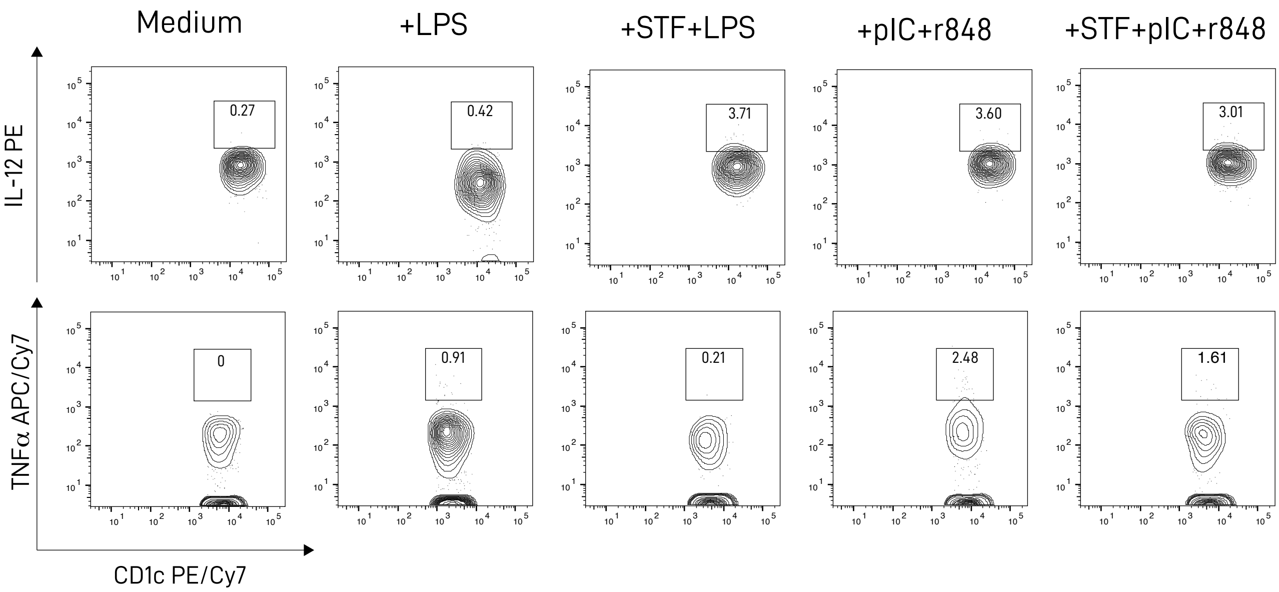

Figure S5. Production of IL-12 by cDC1 under ER stress. *In vitro* OP9-DL1-differentiated dendritic cells were treated with 1 μg/mL Tunicamycin (TM) for 6 hours and Brefeldin A for the last 4h. Production of IL-12 by cDC1 (HLA-DR+ CLEC9A+) was determined by flow cytometry. FACS plots are representative of 3 independent experiments (n=3). The graph shows a pool of 3 different samples from independent experiments, in which each dot represents one sample (n=3). Error bars indicate mean ±SEM. Wilcoxon matched-pairs signed rank test.

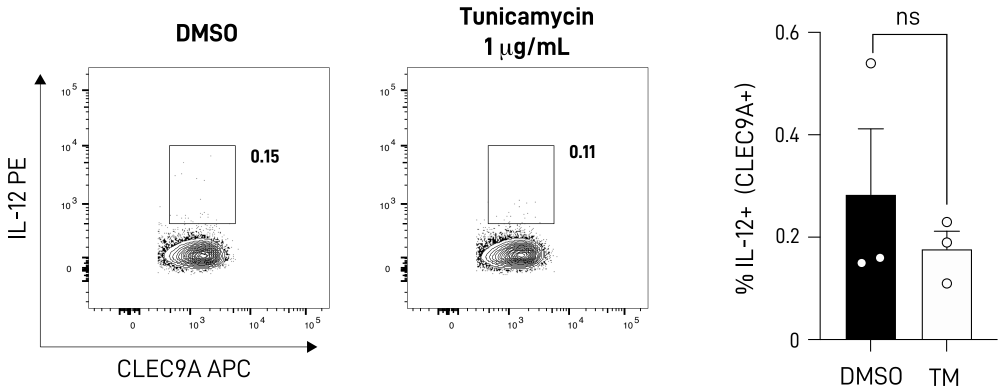

Figure S6. Unprocessed scans of original western blots. IRE1 and BiP protein expression of cDC1 cells was determined through western blot analysis and compared to monocyte-derived DCs (moDCs), CD34+ DC precursors isolated from cord blood as well as total cord blood mononuclear cells (CBMC). Tunicamycin (1mg/ml final concentration, 8hr stimulation) and Thapsigargin (500nM final concentration, 8hr stimulation) were used as ER-stress inducers in CBMC as positive controls. B-actin was used as protein loading control.

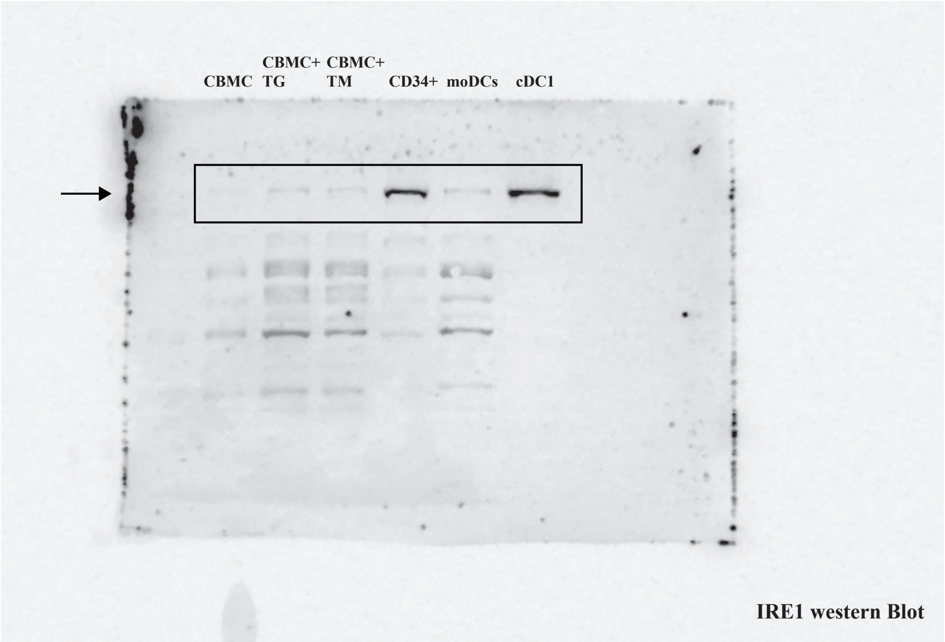

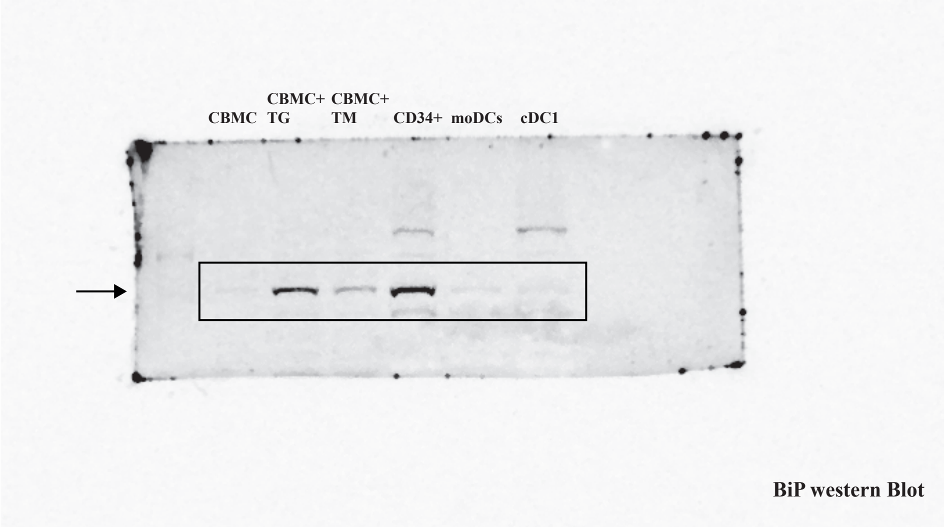

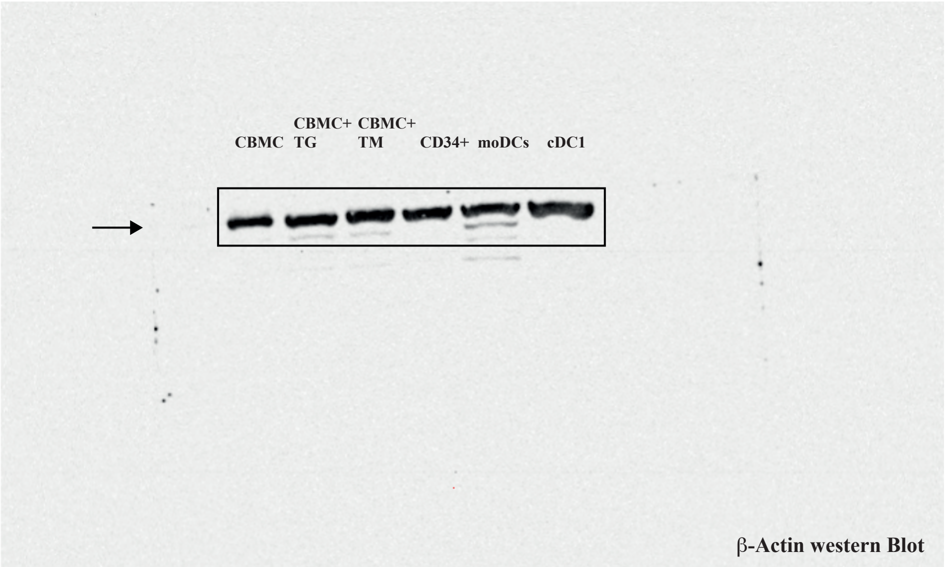

Supplement: Supplementary file 1 — Supporting Information [file EJI-52-1069-s001.pdf]
